# Attention-Based Deep Learning Approach for Breast Cancer Histopathological Image Multi-Classification

**DOI:** 10.3390/diagnostics14131402

**Published:** 2024-07-01

**Authors:** Lama A. Aldakhil, Haifa F. Alhasson, Shuaa S. Alharbi

**Affiliations:** Department of Information Technology, College of Computer, Qassim University, Buraydah 52571, Saudi Arabia; 441212544@qu.edu.sa (L.A.A.); shuaa.s.alharbi@qu.edu.sa (S.S.A.)

**Keywords:** breast cancer, breast tumors, convolutional neural networks, deep learning, diagnosis, transfer learning, histopathological images, image classifier

## Abstract

Breast cancer diagnosis from histopathology images is often time consuming and prone to human error, impacting treatment and prognosis. Deep learning diagnostic methods offer the potential for improved accuracy and efficiency in breast cancer detection and classification. However, they struggle with limited data and subtle variations within and between cancer types. Attention mechanisms provide feature refinement capabilities that have shown promise in overcoming such challenges. To this end, this paper proposes the Efficient Channel Spatial Attention Network (ECSAnet), an architecture built on EfficientNetV2 and augmented with a convolutional block attention module (CBAM) and additional fully connected layers. ECSAnet was fine-tuned using the BreakHis dataset, employing Reinhard stain normalization and image augmentation techniques to minimize overfitting and enhance generalizability. In testing, ECSAnet outperformed AlexNet, DenseNet121, EfficientNetV2-S, InceptionNetV3, ResNet50, and VGG16 in most settings, achieving accuracies of 94.2% at 40×, 92.96% at 100×, 88.41% at 200×, and 89.42% at 400× magnifications. The results highlight the effectiveness of CBAM in improving classification accuracy and the importance of stain normalization for generalizability.

## 1. Introduction

The global incidence of breast cancer reached a concerning rate of 2.3 million diagnosed cases in 2022, tragically claiming the lives of 670,000 individuals [1]. Breast cancer occurs when breast cells mutate and grow uncontrollably. Early detection is vital for effective treatment. Visible indicators that warrant breast cancer screening include a palpable lump in the breast, dimpling in the breast skin texture, redness or swelling of the breast skin, blisters or sores on the breast surface, abnormal nipple discharge, and nipple inversion or flattening [2].

Early and precise diagnosis of breast tumors significantly improves prognosis and is primarily achieved through histopathology. This procedure involves a biopsy, which entails extracting soft tissue samples from suspicious areas using techniques such as fine-needle aspiration, core needle biopsy, vacuum-assisted biopsy, and surgical biopsy [3]. After extraction, the samples are affixed to microscopic slides. Before examination, the tissues undergo staining with hematoxylin and eosin (H&E), which helps distinguish and highlight particular features of the translucent tissue composition and cellular details [4].

Expert pathologists analyze the histological characteristics of the H&E-stained tissue sections, scanning for cancerous cells and abnormalities within the typical structures of the breast throughout the examination process [5]. However, histopathological slides present inherent challenges due to their high complexity and the diversity of tumor tissues. The complexity arises from the pronounced coherency of cancerous cells, substantial intra-class differences, and limited inter-class distinctions within the images. Additionally, images of the same class often exhibit significant resolution variations. Due to this, accurate diagnosis using this type of imaging modality is considered difficult and time consuming [6].

Deep learning (DL) has emerged as a groundbreaking innovation in artificial intelligence. Positioned as a subset of machine learning (ML), the study of DL revolves around training artificial neural networks to discern complex patterns within expansive datasets for various applications. The process of DL mirrors the functioning of the human brain, where interconnected neurons process and store information. DL employs models consisting of multiple layers of processing to acquire data representations at varying levels of abstraction. A crucial characteristic of DL is the ability to swiftly and accurately process copious amounts of data without necessitating explicit programming. As a result, DL models have established new benchmarks for state-of-the-art performance, opening up many possibilities of advancements across various disciplines, including natural language processing, image recognition, and autonomous vehicles [7].

Over the years, DL has seen substantial growth, receiving significant attention in the medical imaging research field [8]. The most popular DL architecture is the convolutional neural network (CNN). CNNs have achieved outstanding results in diagnostic applications of breast cancer from histopathological images [6,9]. These diagnostic applications include tumor detection [10], breast cancer grading [11], breast cancer sub-type classification [12], assessment of tumor heterogeneity and micro-environment [13], and assessment of receptor status and intrinsic sub-type [14].

While numerous DL-based approaches have been proposed in the literature, there is still room for improvement. Among the challenges that negatively affect the generalization of DL approaches for breast cancer diagnosis are the lack of sufficient representative images, the complexity of histology images captured at different magnifications, and the variability in laboratory H&E tissue-staining methods [15,16]. Hence, there is a need to develop an improved approach capable of extracting deep representative features effectively and accurately from breast histopathology images and classifying them into their respective sub-types while utilizing the currently available datasets. This paper aims to address this issue by proposing an attention-based DL approach while utilizing stain normalization and data augmentation techniques to address the diagnostic challenges of multi-class classification of breast tumors, which is crucial for ensuring timely and appropriate patient treatment.

The contributions of our paper are as follows:

1. We proposed the Efficient Channel-Spatial Attention Network (ECSAnet), which is an improved EfficientNetV2 architecture [17]. Our model integrates a convolutional block attention module (CBAM) [18] that efficiently captures features in both the channel and spatial dimensions. It refines the learned features by discerning the most discriminative features, focusing on what is important and where it is located within an image. Moreover, our model incorporates two additional fully connected layers for increased learning capacity.
2. We proposed an approach for creating an oversampled balanced dataset using data augmentation techniques to minimize the data imbalance and increase diversity.
3. We verified that the CBAM, FC layers, and Reinhard stain normalization [19] components helped increase the accuracy and generalization of our proposed model through an ablation study.
4. We compared our proposed model against state-of-the-art models, including AlexNet, DenseNet121, EfficientNetV2-S, InceptionNetV3, ResNet50, and VGG-16. The analysis showed that our model is more accurate than the mentioned models at most magnification settings.

The paper is organized as follows: Section 2 surveys previous studies relevant to the research. Section 3 outlines the key elements of the methodology applied. Section 4 details the results achieved, while Section 5 discusses these results. Section 6 suggests directions for future work. Finally, Section 7 concludes the paper.

## 2. Literature Review

Many studies have previously addressed the essential problem of automated breast cancer classification using DL-based approaches for histopathological images. Generally, the classification of breast histopathological images is approached through two distinct methods: binary and multi-class. Binary classification distinguishes between benign and malignant tumors, while multi-class classification is a more challenging problem than binary classification, as it involves classifying images into distinct tumor sub-types.

In their comprehensive study, Nasser et al. [16] identified CNNs as the leading DL method for detecting breast cancer, making them a popular choice among researchers. Moreover, previous studies have reported that CNNs consistently outperform other methods regarding accuracy within this field. This outstanding performance is primarily due to the CNNs’ innate ability to autonomously extract and classify essential, discriminative features from complex image data. There are two primary approaches to developing CNNs: “de novo”, where the network is built and trained from scratch; and transfer learning, which makes use of CNNs that have been pre-trained on large datasets, such as AlexNet, ResNets, GoogLeNet, or VGGNets, as a baseline [16].

The significance of transfer learning approaches lies in their potential to avoid expensive data-labeling efforts and enhance learning performance by leveraging knowledge from related domains [20]. Among the studies that have applied transfer learning for breast cancer histopathological images are Yari et al.’s [21]. They used two pre-trained models: ResNet50 and DenseNet-121. They modified the last FC layers in the models differently for each classification type. Consequently, they set the number of output features to eight for multi-class and two for binary classification. They evaluated the models for magnification-dependent (MD) and -independent (MI) classification. The results in binary classification demonstrated high image-level accuracy with values ranging from 99.02% to 100% for MD binary classification and 99.26% for MI binary classification. Regarding multi-class classification, the approach yielded MD accuracies ranging from 94.95% to 97.96% and 95.57% for MI classification.

Similarly, another study by Boumaraf et al. [22] used the ResNet18 pre-trained model for binary and multi-class classification, distinguishing between MD and MI classification. Following a block-wise fine-tuning strategy, they adapted the model to the BreakHis dataset. Furthermore, they modified the ResNet18 classifier by incorporating an additional FC layer for more robust feature learning. Their reported results showcased high image-level average accuracies of 98.84% and 92.15% for MD binary and eight-class classification, respectively. As for MI classification, it achieved 98.42% and 92.03% accuracy for binary and eight-class classification, respectively.

To minimize the feature complexities of varied magnifications in histopathological images, Sheikh et al. [12] proposed a multi-scale input and multi-feature network (MSI-MFnet). Their proposed approach captures the tissue cells and texture features by fusing multi-scale hierarchical feature maps from different layers using image patches. MSI-MFnet was evaluated for both binary and multi-class classification using two datasets: BreakHis and ICIAR2018. The results highlighted the importance of fusing multi-scale inputs and the need to effectively use multi-feature maps to extract distinctive salient features while preserving coarse-scale features. For the ICIAR2018 dataset, they achieved patch-wise accuracies of 82% and 68% for binary and multi-class classifications, respectively. Moreover, for the BreakHis dataset, they achieved patch-wise accuracies of 98% and 87% for binary and multi-class classification, respectively.

The effectiveness of attention mechanisms in enhancing the performance of computer vision methods has garnered substantial empirical support in recent years. Fundamentally, attention mechanisms selectively concentrate on salient features within an image, similar to the human visual system’s capacity to discern and prioritize regions of interest in complex visual scenes. These mechanisms can be characterized as dynamic selection processes that adaptively adjust weights based on the significance of different features [23].

Given their efficiency, attention mechanisms have been adopted into DL-based approaches for classifying histopathological images of breast cancer. For instance, Togacar et al. [24] incorporated a CBAM attention mechanism in their BreastNet architecture. The CBAM block was used to refine features adaptively by processing feature maps across spatial and channel dimensions, which enhanced model accuracy and computational efficiency. In addition to CBAM, BreastNet integrated various components, such as convolutional, dense, and residual blocks, alongside a hyper-column technique. Each component improved feature discrimination, strengthened the gradient flow, optimized feature selection, and lastly, enabled multi-scale analysis through the hyper-column approach. In comparative assessments against state-of-the-art models like AlexNet, VGG-16, and VGG-19, BreastNet showed improved performance, achieving accuracies ranging from 95.88% to 98.52% at different magnification factors.

Another study by Li et al. [25] proposed an approach of adapting the squeeze-and-excitation (SE) attention mechanism into the DenseNet121 architecture, which they referred to as the interleaved DenseNet with SENet (IDSnet). Additionally, their approach addresses computational resource constraints and network overfitting by incorporating global average pooling, which reduces the model’s complexity. IDSnet uses a pre-trained and fine-tuned DenseNet-121 to extract feature maps from histopathological images, which are then refined using SENet modules to capture essential channel-wise global information. They evaluated IDSnet classification performance compared to VGG-16 and ResNet50 models, where it proved superior, achieving patient recognition rates between 84.6% and 90% and image recognition rates from 84.5% to 89.1% across different magnifications.

Zou et al. [26] utilized the efficient channel attention (ECA) module within their proposed attention high-order deep network (AHoNet), which enhances the ResNet18 architecture. With an integrated ECA module, their approach incorporated non-dimensionality reduction and local cross-channel interaction. In addition, they implemented matrix power normalization to compute second-order covariance statistics, which further improved AHoNet’s ability to capture salient local and global features effectively. The AHoNet model was evaluated on two datasets: BreakHis and BACH. Using BreakHis, they achieved impressive accuracies of 99.09% at the image level and 99.29% at the patient level. Whereas using BACH, it achieved an accuracy of 85%.

## 3. Materials and Methods

**Figure 1:**
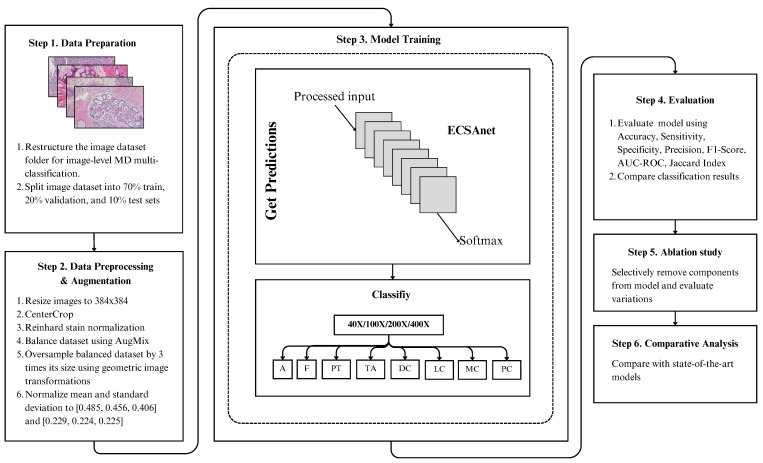
Workflow of the approach employed for breast histology image classification. Our approach begins with dataset processing steps, followed by training the ECSAnet model to extract features and obtain classification predictions, after which we move on to model evaluation and analysis steps.

This section will provide details of the study methodology, covering (i) an overview of CNNs, (ii) the EfficientNetV2 architecture, (iii) the CBAM attention mechanism, (iv) the proposed approach, (v) the dataset employed, (vi) the data pre-processing and augmentation techniques used, (vii) the experimental setup, and finally, (viii) the evaluation metrics that we have used. Figure 1 presents a summary of the study workflow.

### 3.1. Convolutional Neural Networks

**Figure 2:**
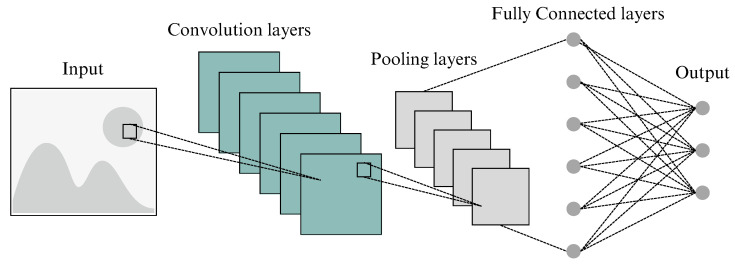
Basic architecture of a CNN [28].

The CNN architecture is a widely recognized DL architecture with diverse applications in computer vision, such as image classification, action recognition, pose estimation, object detection and tracking, text detection and recognition, and many more [27]. The CNN architecture has seen the development of various variants, including well-known ones like LeNet, AlexNet, and VGGNet, among others. Although specific architectures may introduce additional layers or modifications, the fundamental structure of a CNN can be said to consist of three layers: a convolutional layer, a pooling layer, and a fully connected layer [27,28]. Figure 2 illustrates the basic architecture of a CNN.

- The convolutional layer: This layer plays a major role in a CNN, as its name suggests. It includes convolution kernels, also called filters, which are responsible for generating feature maps. Each neuron within a feature map establishes connections with a neighboring region of neurons in the preceding layer. Through this process, the convolutional layer can learn representations from the spatial dimensions inherent in the input data [27].
- The pooling layer: This layer reduces the dimensionality of feature maps to decrease the number of parameters and computational complexity. Typically positioned between convolutional layers, each feature map within the pooling layer establishes connections with the corresponding feature map in the preceding convolutional layer [28].
- The fully connected layer: This layer helps capture complex relationships between the features and make predictions based on the learned representations. In this layer, each neuron is connected to every neuron in the preceding layer. Consequently, the output of each neuron in the previous layer is treated as an input for every neuron within the fully connected layer [27].

### 3.2. EfficientNetV2

**Figure 3:**
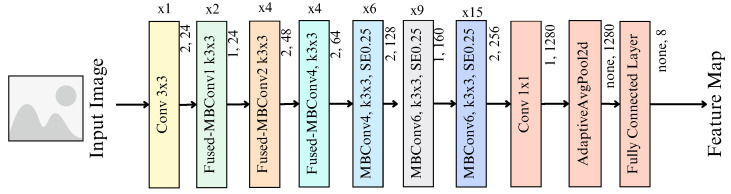
Architecture of EfficientNetV2 [17].

EfficientNetV2 [17] is an improved network of models (EfficientNetV2-S/M/L) built upon EfficientNet [29]. A distinctive characteristic of the EfficientNet architecture lies in its implementation of a compound scaling method. This method allows all model layers to be uniformly and effectively scaled by a constant ratio, optimizing the depth, width, and resolution of the layers for any resource constraint. Consequently, the models can allocate their computational resources to focus on more salient regions within an image, facilitating the learning of fine-grained features and ultimately enhancing accuracy. This scaling approach is achieved while maintaining fewer parameters and floating point operations, ensuring a favorable trade-off between model efficiency and performance. EfficientNetV2 improves upon EfficientNet in three ways. First, EfficientNetV2 enhances training speed by adopting a progressive learning technique. This technique dynamically adjusts the regularization parameters with the image size during training. Secondly, the architecture of EfficientNetV2 incorporates fused mobile inverted bottleneck convolution (Fused-MBConv) layers to replace the early layers. This replacement serves to enhance the training speed further. Thirdly, EfficientNetV2 introduces a modified scaling rule that constrains the maximum image size. This limitation mitigates the substantial memory consumption associated with larger image sizes. As a result, the training speed is improved, and computational resources are utilized more efficiently. Collectively, EfficientNetV2 excels in multiple dimensions in comparison to its predecessors. It surpasses previous models in terms of training speed, parameter efficiency, and overall accuracy. The architecture of EfficientNetV2 can be seen in Figure 3.

### 3.3. Convolutional Attention Block Module

The attention mechanism used in this paper is the CBAM. This module was first introduced in [18]. It is an efficient attention mechanism designed to work with feed-forward CNNs. This module combines the representative power of both channel attention (CAM) and spatial attention (SAM) modules with a small overhead. Essentially, it applies a CAM and SAM sequentially to focus on learning “what” and “where” to pay attention to along the channel and spatial dimensions. This process can efficiently learn salient features and suppress irrelevant ones.

**Figure 4:**
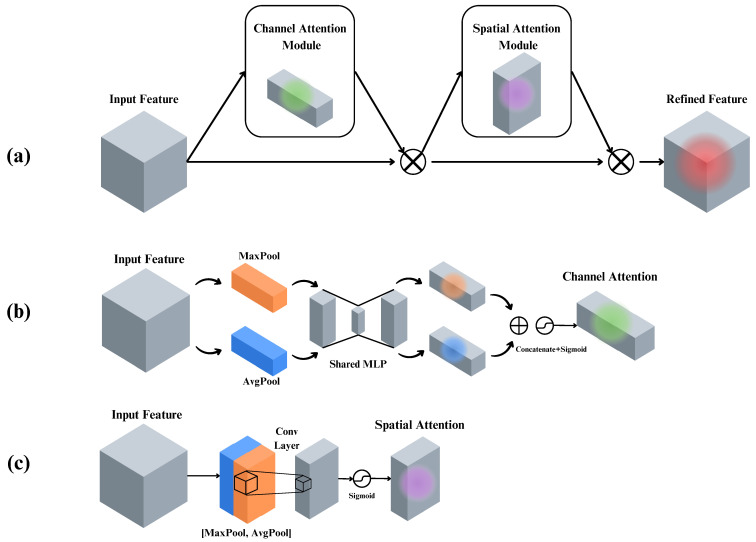
Computation processes of the attention modules: (**a**) CBAM, (**b**) CAM, and (**c**) SAM [18].

When given a feature map tensor $F\in R^{C\times H\times W}$, the CBAM module sequentially computes a 1D channel attention map $M_{c}\in R^{C\times 1\times 1}$ and a 2D spatial attention $M_{s}\in R^{1\times H\times W}$; in summary, the attention is computed using the following equation:

(1) $$\begin{array}{c} F^{\prime }=M_{c}\left(F\right)⊗F\\ F^{\prime \prime }=M_{s}\left(F^{\prime }\right)⊗F^{\prime } \end{array}$$

where ⊗ indicates element-wise multiplication and $F^{\prime \prime }$ is the final refined feature map. Figure 4 depicts an overview of the computations of the CBAM and its sub-modules, SAM and CAM.

### 3.4. Proposed Approach

An improved model called ECSAnet is proposed to effectively extract discriminative features from histology images for breast cancer classification. ECSAnet adopts the EfficientNetV2-S architecture as its baseline. ECSAnet improves upon the baseline model in three ways:

1. It has an added CBAM block placed before the final classifier block.
2. The final classifier block has two additional fully connected layers.
3. The number of output features of the last FC layer is adjusted from 1000 to 8 to align with the eight tumor sub-types in the BreakHis dataset.

**Figure 5:**
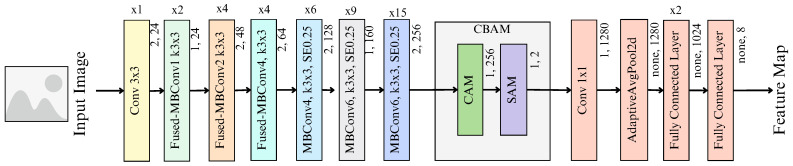
Architecture of ECSAnet.

Following these changes, the ECSAnet model architecture summary is listed in Table 1 and illustrated in Figure 5.

**Figure 6:**
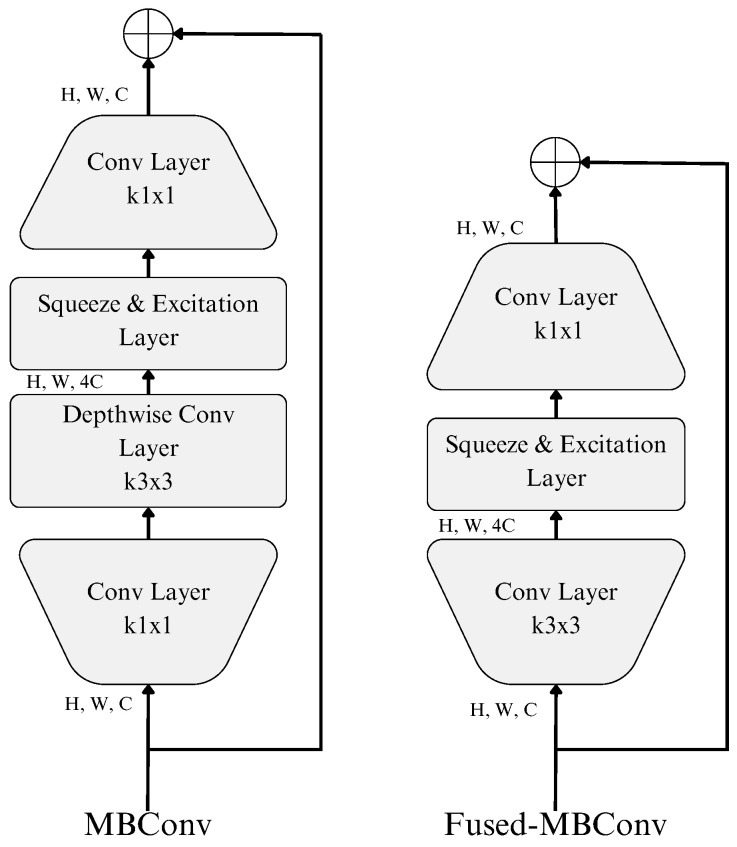
Structures of MBConv and Fused-MBConv [17,30,31].

The main building blocks of the architecture are the MBConv [30] and the Fused-MBConv [31] layers. The MBConv is a complex convolutional layer consisting of a squeeze-and-excitation module and a depth-wise convolutional layer. Meanwhile, the Fused-MBConv replaces the depth-wise convolutional layer with a 3 × 3 convolutional layer. The structures of these two layers are shown in Figure 6.

The addition of FC layers in deep learning models is particularly useful for tasks that require complex relationships between inputs and outputs. FC layers connect all neurons between layers, allowing them to use all the information contained in the input layer. This makes FC layers a powerful tool for enhancing machine learning models, as they are capable of accurately identifying the complex relationships in the input data. However, this type of layer is more difficult to train than other layers because it is computationally inefficient. This issue can be solved by combining it with CBAM.

CBAM is designed to enhance convolutional neural networks by focusing on the most relevant parts of the input data. This selective focus enables networks to better handle large images or images containing complex details, which ultimately improves performance by refining the learned feature representations. Due to this, CBAM can be used to potentially reduce the amount of training time required with fully connected layers. Moreover, CBAM reduces the computational cost of training a network by avoiding the necessity of processing the entire input image each time.

The combination of CBAM with additional fully connected layers leverages the strengths of both components, which include CBAM’s enhanced feature refinement capability and reduced model overhead and the FC layer’s capability to identify complex relationships to improve model accuracy. The components can work synergistically as CBAM refines the features and reduces the amount of irrelevant information processed by the subsequent FC layers. Then, the additional FC layers add increased learning capacity, allowing them to learn and model complex relationships within refined feature representations more effectively. Additionally, the computational complexity associated with FC layers can be mitigated with fewer, more informative features.

### 3.5. Dataset

The dataset employed in this work is the Breast Cancer Histopathological Image Classification (BreakHis) dataset [32], which is publicly accessible online at this link: https://web.inf.ufpr.br/vri/databases/breast-cancer-histopathological-database-breakhis/ (accessed on 1 Decamber 2023). This dataset comprises a total of 9109 microscopic images depicting breast tumor tissue. The images were collected from 82 patients and were captured at varying magnifications, specifically 40×, 100×, 200×, and 400×. Each image is stored in the PNG format and possesses the following specifications: a resolution of 700 × 460 pixels, a 3-channel RGB color space, and an 8-bit depth in each channel.

**Figure 7:**
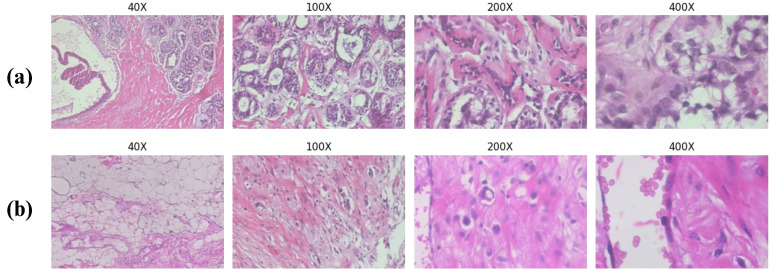
(**a**) Samples for benign tumor tissues; (**b**) samples for malignant tumor tissues [32].

The BreakHis dataset is divided into two main groups: benign and malignant. Each group is then divided into sub-types. Benign tumor tissues are divided into four tumor classes: adenosis (A), fibroadenoma (F), phyllodes tumor (PT), and tubular adenoma (TA). On the other hand, malignant tumor tissues are divided into four tumor classes: ductal carcinoma (DC), lobular carcinoma (LC), mucinous carcinoma (MC), and papillary carcinoma (PC). Samples of a benign tumor and malignant tumor are shown in Figure 7. The class distribution of the BreakHis dataset is shown in Table 2.

The dataset has been reorganized for magnification-dependent image-level multi-classification and split into three parts: 70% for training, 20% for validation, and 10% for testing.

### 3.6. Data Pre-Processing and Augmentation

Effectively preparing and enhancing data is a key contributor to successful DL models, particularly when dealing with task-specific requirements. In the context of breast histopathology and medical imagery, augmentation strategies must preserve essential image characteristics while introducing a diverse range of variations. This diversity is necessary to ensure models can generalize well when deployed in real-world diagnostic scenarios. Clinicians can interpret histopathology images irrespective of changes in angle, orientation, or scale, and this robustness should also be characteristic of DL models applied to the same task.

As outlined in Algorithm  1, three functions are present: pre-process, balance, and transform.
**Algorithm 1** Constructing an oversampled balanced dataset**Input:** main_dir: Directory containing class folders with images**Output:** Oversampled balanced dataset  1:Identify and enumerate all classes in main_dir  2:Count the number of images per class  3:max_class_count← maximum image count across classes  4:oversample_target←3×max_class_count  5:**for** each class *c* **do**  6:    image_count← count of images in class *c*  7:    **if** image_count<oversample_target **then**  8:        Oversample class *c* up to oversample_target using transform  9:    **end if**10:    **for** i←1 to oversample_target **do**11:        index←(i−1)modimage_count12:        image_path← path of index-th image in class *c*13:        Load image from image_path14:        Apply preprocess to the image15:        **if** image_count<max_class_count **then**16:           Apply balance to augment the image17:        **end if**18:        Apply transform to the image19:        Return image and class label20:    **end for**21:**end for**

**Figure 8:**
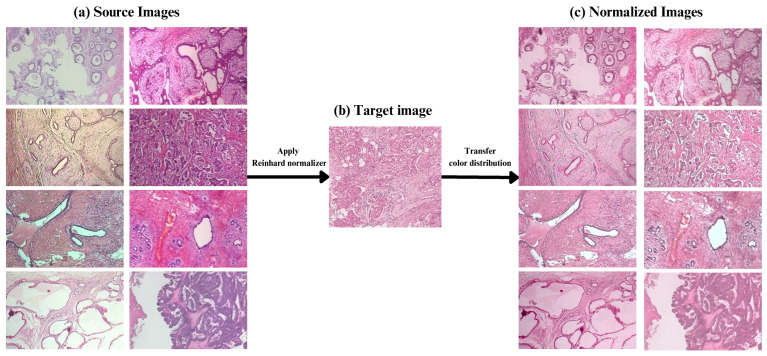
Demonstration of Reinhard stain normalization on breast histopathology images. (**a**) Source images before normalization. (**b**) The target image providing the reference color distribution. (**c**) Images after applying the Reinhard method for color normalization.

First, we have the pre-processing function, which is applied to all images, resizing and cropping them to dimensions of 384×384 pixels. To address variations in histopathological image staining, we employ the Reinhard color normalization technique [19]. This method adjusts the color distribution of a source image to match that of a target image, as expressed in Algorithm  2. Selecting an appropriate target image for this procedure requires expertise in the domain; therefore, we rely on an image identified in a previous study [33]. In that study, the authors collaborated with a pathologist to select a suitable image from the Mitosis-Atypia database [34]. For visual reference, the target image and an illustration of the stain normalization process are presented in Figure 8.
**Algorithm 2** Reinhard Color Normalization**Input:** Source image $I_{s}$ in RGB, Target image $I_{t}$ in RGB**Output:** Normalized source image $I_{s}^{\prime }$ in RGB  1:Convert $I_{s}$ from RGB to Lab color space to get $L_{s},a_{s},b_{s}$  2:Convert $I_{t}$ from RGB to Lab color space to get $L_{t},a_{t},b_{t}$  3:Compute mean $\mu_{L_{s}}$, $\mu_{a_{s}}$, $\mu_{b_{s}}$ and standard deviation $\sigma_{L_{s}}$, $\sigma_{a_{s}}$, $\sigma_{b_{s}}$ of $I_{s}$  4:Compute mean $\mu_{L_{t}}$, $\mu_{a_{t}}$, $\mu_{b_{t}}$ and standard deviation $\sigma_{L_{t}}$, $\sigma_{a_{t}}$, $\sigma_{b_{t}}$ of $I_{t}$  5:**for** each channel C∈{L,a,b} **do**  6:    Subtract mean from source: $C_{s}^{\prime }\leftarrow C_{s}-\mu_{C_{s}}$  7:    Scale by standard deviation ratio: $C_{s}^{\prime \prime }\leftarrow C_{s}^{\prime }\times \left(\sigma_{C_{t}}/\sigma_{C_{s}}\right)$  8:    Add target mean: $C_{s}^{\prime \prime \prime }\leftarrow C_{s}^{\prime \prime }+\mu_{C_{t}}$  9:**end for**10:Merge normalized channels to get Lab image $I_{s}^{\prime }\leftarrow \left\{L_{s}^{\prime \prime \prime },a_{s}^{\prime \prime \prime },b_{s}^{\prime \prime \prime }\right\}$11:Convert $I_{s}^{\prime }$ from Lab to RGB color space

Next, we have the balancing function, which aims to equalize the number of samples across all classes. To achieve this, we oversample the minority classes to match the class with the maximum number of samples. This approach utilizes the AugMix [35] image augmentation technique, which produces new, augmented images. This process involves blending several images that have undergone traditional transformations, such as rotation and posterizing. The specific blending weights for each combination generated by AugMix are sampled from a Dirichlet distribution. The advantage of using AugMix is that it allows for the creation of a wide range of image variations. At the same time, it preserves the essential semantic features of the original images, resulting in more robust variations [35].

Finally, in order to artificially increase the training set, traditional augmentations are used to create modified versions of the images. This is particularly important for deep learning models, where the performance is highly dependent on the size and quality of the training data. Thus, we have the transform function, which serves to triple the size of the balanced image classes by geometrically augmenting their respective images using the following transformations:

- Random flips: Images are flipped horizontally and vertically with a probability of 0.5.
- Random rotations: Affine transformations include rotations at random angles from −45 to 45 degrees.
- Random translations: Images are translated vertically and horizontally by up to 10%.
- Random scaling: The size of the images is randomly adjusted by a scaling factor between 0.8 and 1.2.
- Random shearing: Images are sheared at random angles ranging from 0 to 10 degrees.

We also normalize the image tensors in this function to ensure standardized input data. This process adjusts the pixel values in each channel to have a standardized mean and standard deviation. Image normalization is an essential pre-processing step to stabilize the training process and increase convergence speed. When given an image tensor X with dimensions (H,W,C), in addition to a sequence of mean values $\mu =(\mu_{1},\mu_{2},\ldots ,\mu_{C})$ and standard deviation values $\sigma =(\sigma_{1},\sigma_{2},\ldots ,\sigma_{C})$, the normalized image tensor is computed as follows:

(2) $$\begin{array}{c} X^{\mathrm{norm}}\left[h,w,c\right]=\frac{X\left[h,w,c\right]-\mu_{c}}{\sigma_{c}} \end{array}$$

In this equation, X[h,w,c] denotes the original pixel value at height *h*, width *w*, and channel *c*. The term $\mu_{c}$ represents the mean of the pixel values for channel *c*, while $\sigma_{c}$ stands for the standard deviation of the pixel values for channel *c*. The normalized tensor pixel values, $X^{\mathrm{norm}}\left[h,w,c\right]$, are acquired by subtracting the mean $\mu_{c}$ from the original value X[h,w,c], and then, dividing the result by the standard deviation $\sigma_{c}$.

The rationale behind using the two separate functions, balance and transform, is to address two issues in the dataset: class imbalance and limited data. The balance function allows us to apply a more aggressive augmentation strategy specifically to the minority classes, effectively increasing their representation in the dataset without introducing excessive duplication of the existing samples. The transform function then applies a consistent set of geometric transformations across the entire dataset, further expanding the diversity of the data and helping the model to generalize. This approach ensures that class imbalance is mitigated while the overall dataset benefits from a broad range of variations, reflecting the complexities of real-world data.

**Figure 9:**
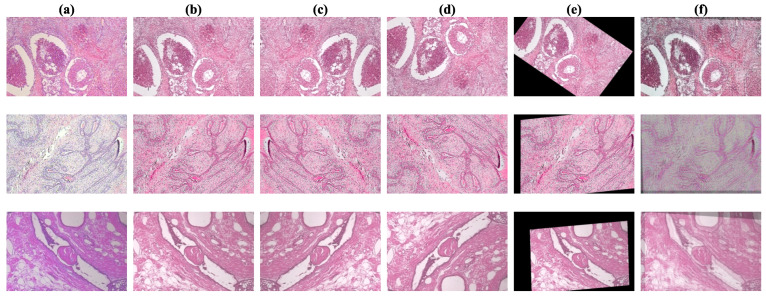
Demonstration of implemented data augmentations. Column (**a**) shows the images in their original state. Column (**b**) shows the images after stain normalization. Columns (**c**,**d**) show the images after horizontal and vertical flips, respectively. Column (**e**) shows the images after random affine transformations, which include scaling, rotation, and translation. Lastly, column (**f**) shows the images after AugMix augmentations.

Some examples of the image augmentations that have been implemented are illustrated in Figure 9.

After applying the data augmentation approach for balancing and oversampling, the resulting training set size is presented in Table 3.

### 3.7. Experimental Setup

All experiments were conducted on Google Colaboratory, a cloud-based service that offers computing resources through a hosted Jupyter Notebook environment built on the Python3.12.0 3 Google Compute Engine backend. A Colab Pro subscription was used to gain access to longer runtimes. The runtime resources used were a T4 GPU with 15 GB RAM, 12.7 GB system RAM, and 201.2 GB of disk space. Additionally, Colab enables easy integration of Google Drive, which was used to store and access the dataset, model weights, and log files. The code for this paper is available in the GitHub repository at this link: https://github.com/LamaAldakhil/ECSAnet (accessed on 1 June 2024).

The following sections will specify the Python libraries and training hyperparameters used in our proposed approach.

#### 3.7.1. Python Libraries Used

This research relies on several Python libraries that provide tools for DL and data analysis. Listed in Table 4 are the libraries used, in addition to a brief description of their functions that were relevant to our research.

#### 3.7.2. Model Fine-Tuning

The PyTorch library was utilized to build the DL models. PyTorch is a well-known library in the DL community and is widely used for many computer vision applications. It is a well-rounded library encompassing all the tools necessary for designing, building, and training DL models. Through this library, we built our pre-trained models, including our main baseline model, EfficientNetV2-S. To develop our proposed model, we created a subclass of the EfficientNetV2-S architecture. This approach enables building upon the existing model, incorporating the proposed improvements, and fine-tuning it for the task involving eight image classes.

Additionally, transfer learning was employed. Transfer learning reduces resource usage and training time compared to training from scratch. Moreover, it enables the model to effectively learn low-level features such as edges, textures, and colors, enhancing performance and minimizing the challenges associated with limited image data. The pre-trained weights were sourced from a model originally trained on the ImageNet dataset, which consists of 1000 image classes and contains over 14 million samples.

#### 3.7.3. Training Hyperparameters

In ML, the performance of algorithms depends significantly on the training hyperparameters. We initiated the experiments with a batch size of 16 images. This number balances computational load and training efficiency, as it requires less memory than larger batches, making it suitable in environments with limited computing resources.

To avoid overfitting, where a model becomes too closely fitted to the training data and less able to generalize, we limited the training to 50 epochs. Additionally, an early stopping mechanism was used to halt training if the validation loss did not improve after 25 epochs, thereby preserving computational resources and model integrity.

For calculating loss during training and validation, we used the cross-entropy loss function as a criterion. In a multi-class classification task, this function is calculated using the following formula:

(3) $$\begin{array}{c} CrossEntropyLoss=-\sum_{c=1}^{M}y_{o,c}\log\left(p_{o,c}\right) \end{array}$$

Here, *M* is the number of class categories, $y_{o,c}$ indicates the presence or absence of class *c* in observation *o*, and $p_{o,c}$ is the predicted probability of observation *o* belonging to class *c*.

As an optimizer for the loss function, stochastic gradient descent (SGD) was selected. SGD updates model parameters by calculating the gradient of the loss function based on a random data subset. The model’s weights are adjusted iteratively according to the following rule:

(4) $$\begin{array}{c} w_{t+1}=w_{t}-\gamma_{t}\nabla_{w}Q\left(z_{t},w_{t}\right) \end{array}$$

In this equation, *w* represents the weights that are updated by a time step t+1 by taking the previous weights $w_{t}$ and adjusting them in the direction opposite to the gradient of the loss function at the current step. This adjustment is scaled by a factor of $\gamma_{t}$, which is the learning rate at time *t*. The gradient, denoted as $\nabla_{w}Q\left(z_{t},w_{t}\right)$, reflects the direction and magnitude of the steepest ascent of the loss function *Q* with respect to the weights, given the current input $z_{t}$ [40].

The learning rate $\gamma_{t}$ set for SGD is 0.001. This is a value used commonly for adapting pre-trained models that can help prevent rapid losses of previously learned information and increases in error rates.

Lastly, the ReduceLROnPlateau was chosen as a learning rate scheduler based on its effectiveness in decreasing the learning rate when progress stalls. It observes the validation loss and, after a specified ’patience’ period without improvement, reduces the learning rate by a factor of 0.1, aiding the model’s convergence toward better performance.

Table 5 gives a summary of the selected hyperparameters.

### 3.8. Evaluation Metrics

In our experiments, we assessed the different DL models using various evaluation metrics. These metrics are crucial for understanding various aspects of model performance, including precision, accuracy, F1 score, sensitivity, and specificity. Additionally, we utilize visual tools such as the ROC curve and its associated AUC value to gauge the model’s discriminative ability. The Jaccard index and the confusion matrix provide further insights into the model’s classification capabilities. The following is a detailed breakdown of each evaluation metric, along with their mathematical equations, if present:

- Precision: This performance metric evaluates the precision of predictions made by a model by computing the ratio of true positives to the total number of positive predictions made by the model, as shown in the following equation:
  (5) $$\begin{array}{c} Precision=\frac{TP}{TP+FP} \end{array}$$
- Accuracy: This metric measures the extent to which instances within a dataset are correctly classified, representing the ratio of accurately classified instances to the total number of instances in the dataset, as shown in the following equation:
  (6) $$\begin{array}{c} Accuracy=\frac{TP+TN}{TP+TN+FP+FN} \end{array}$$
- Sensitivity: This metric represents the true positive rate and evaluates a classification model’s ability to identify positive instances correctly. It is equal to the recall metric, and it is also calculated using the same equation as recall:
  (7) $$\begin{array}{c} Sensitivity=Recall=\frac{TP}{TP+FN} \end{array}$$
- Specificity: This metric represents the true negative rate and evaluates the model’s capability to identify negative instances correctly. It is calculated using the following equation:
  (8) $$\begin{array}{c} Specificity=\frac{TN}{FP+TN} \end{array}$$
- F1 score: This metric provides the harmonic mean of precision and recall to comprehensively evaluate the model’s ability to achieve both high precision and recall simultaneously. It is calculated using the following equation:
  (9) $$\begin{array}{c} F1=\frac{2\times Precision\times Recall}{Precision+Recall}=\frac{2\times TP}{2\times TP+FP+FN} \end{array}$$
- AUC-ROC: The ROC curve is a graphical representation that depicts the association between the true positive rate (Sensitivity) and the false positive rate (1−Specificity) at various classification thresholds. Meanwhile, the AUC is a scalar value that summarizes the overall performance of a classifier based on the ROC curve. Ranging from 0 to 1, a higher AUC indicates superior classifier performance. AUC is calculated as the area under the Sensitivity-(1−Specificity) curve.
- Jaccard index: This metric, which is also called the Jaccard similarity coefficient, measures the similarity between two sets by comparing a set of predicted labels for a sample to the corresponding set of labels in another sample [41]. The following equation calculates the similarity of two sets *U* and *V*:
  (10) $$\begin{array}{c} Jaccard\left(U,V\right)=\frac{\left|U\cap V\right|}{\left|U\cup V\right|} \end{array}$$
- Confusion matrix: The evaluation of an ML model’s accuracy often involves the utilization of a confusion matrix, particularly in the context of classification problems. This matrix proves particularly valuable when dealing with imbalanced datasets [42]. Confusion matrices visually represent predicted versus true class labels by plotting instances of each category along matrix rows and columns. Row entries represent actual data classes, while column entries denote predicted classes, offering insights into true positives, false negatives, false positives, and true negatives.

## 4. Results

This section will present the quantitative results, the ablation study, and the comparative analysis.

### 4.1. Quantitative Results

**Figure 10:**
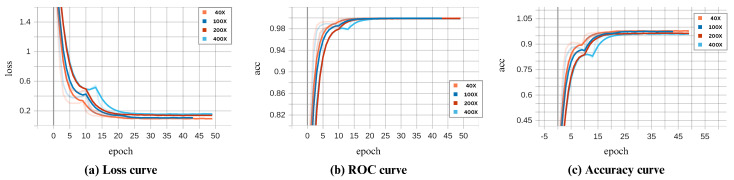
ECSAnet performance on the training set across magnification factors.

**Figure 11:**
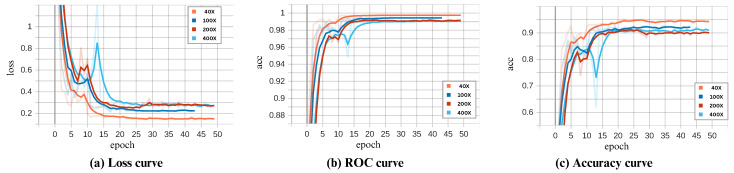
ECSAnet performance on the validation set across magnification factors.

In this paper, we proposed the ECSAnet model for the classification of multi-class breast histopathological images using the BreakHis dataset. The performance curves during model training and validation are illustrated in Figure 10 and Figure 11. As seen in the figures, the model’s performance during training reaches convergence around the 25th epoch and remains consistent thereafter. The dips observed in the validation curves are addressed by a learning rate reduction, which consequently improves performance in the following epochs. The highest validation accuracies, coupled with the lowest losses, were 94.2%, 91.8%, 91.5%, and 91.7% for the 40×, 100×, 200×, and 400× magnifications, respectively.

Whereas the highest testing accuracies achieved were, 94.2%, 92.96%, 88.41%, and 89.42% for 40×, 100×, 200×, and 400× magnification factors, respectively.

The model exhibited excellent generalization on the 40× and 100× magnifications test set. However, it experienced accuracy reductions of 3.09% and 2.28% for the 200× and 400× magnifications, respectively.

**Figure 12:**
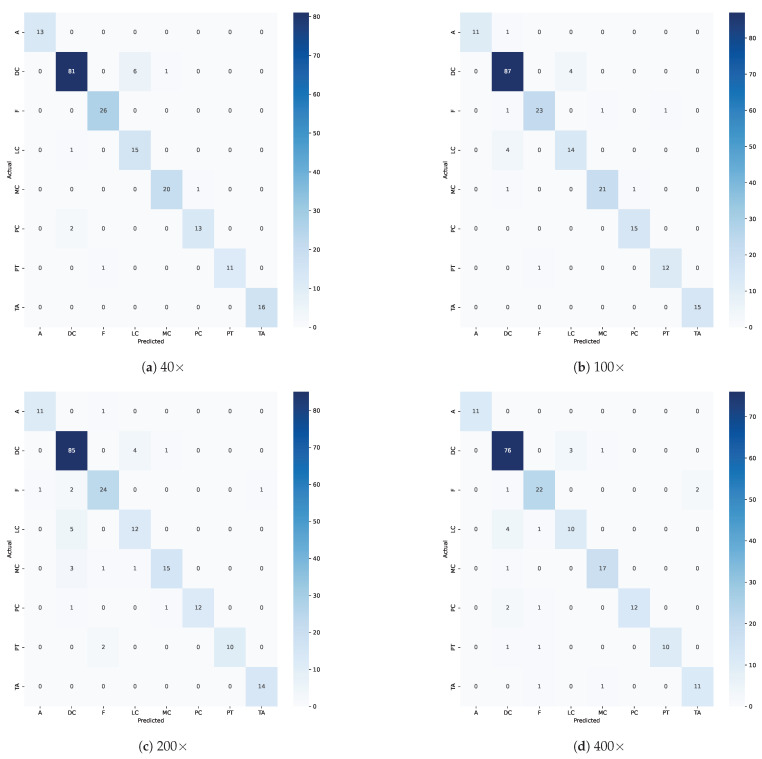
Test set confusion matrices for ECSAnet across magnification factors.

In Table 6, Table 7, Table 8 and Table 9, the ECSAnet classification results using the test set are listed for each breast tissue sub-type and each magnification factor. Additionally, the confusion matrices for the classification are depicted in Figure 12.

**Figure 13:**
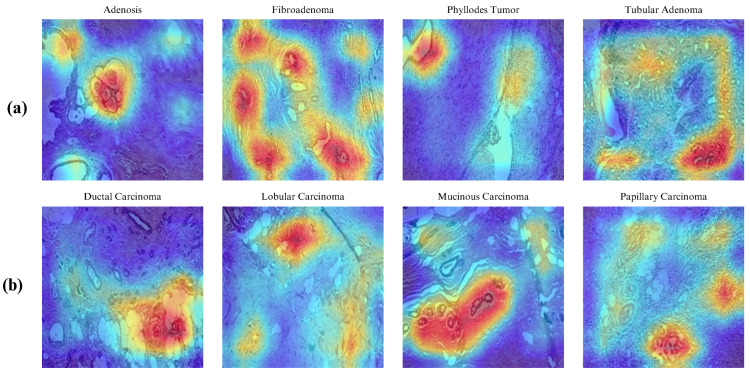
Regions of interest identified by the model using Grad-CAM. (**a**) shows example images of benign classes while (**b**) shows examples for the malignant classes.

Finally, we employed gradient-weighted class activation mapping (Grad-CAM) to generate visual explanations highlighting the regions within breast tissue images deemed most significant by the model in predicting tumor sub-types. Representative samples of these Grad-CAM visualizations are presented in Figure 13. Utilizing this technique, we can interpret the decision-making process of the model, which is particularly crucial in the classification of breast histopathology images.

### 4.2. Ablation Study

To understand the effects and interactions between the components we have added to ECSAnet, we conducted an ablation study. In this study, we conducted five experiments on the 40× magnification data, in which we achieved the best results. The five experiments were (i) ECSAnet without data augmentation, (ii) ECSAnet without balancing data augmentation, (iii) ECSAnet without the addition of the CBAM block, (iv) ECSAnet without the additional FC layers, and (v) ECSAnet without applying stain normalization as a pre-processing step.

**Figure 14:**
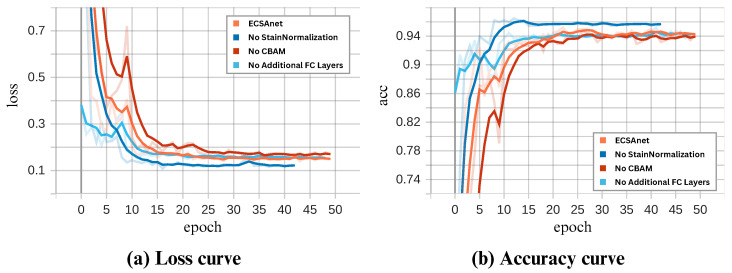
Comparison of the ECSAnet’s performance curves against variations with removed elements.

The validation performance of each approach during training is compared to the ECSAnet model in Figure 14.

In addition, the metrics achieved are shown in Table 10, demonstrating each approach’s performance on the test set.

The results indicate that without applying any data augmentation to the dataset during the training process, the model performance suffers drastically, by 17.87%. This shows that the data augmentation strategy was effective in reducing overfitting and increasing model prediction accuracy.

Subsequently, training the model without class-balancing augmentation showed a small decrease in accuracy, by 0.96%.

Additionally, we have found that adding FC layers to the model structure complements the CBAM block, enhancing the base model’s accuracy in classifying breast tissue images. Specifically, the CBAM block improved the model’s capability to select the most informative features, while the FC layers increased the model’s learning capacity, allowing it to learn refined features more effectively. This synergy is evident by a notable reduction in accuracy when either component is excluded.

Without applying stain normalization, the model showed a validation accuracy of 95.5% during training, a value higher than we saw when we applied it, which was 94.2%. However, the impact of stain normalization becomes more apparent in the test set, where the normalized approach sustained its accuracy, while the non-normalized approach experienced a decrease of 2.75%. In this case, the stain normalization increased the model’s generalization ability.

### 4.3. Comparative Analysis

**Figure 15:**
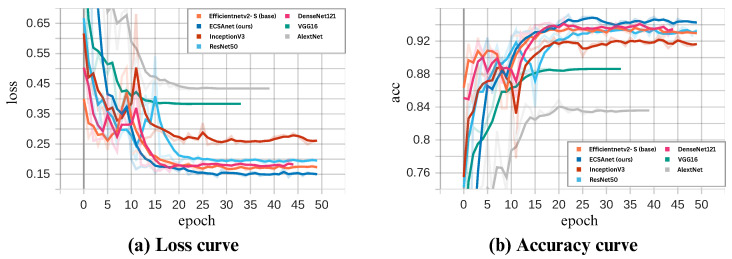
ECSAnet validation performance compared to other state-of-the-art models on the 40× magnification.

**Figure 16:**
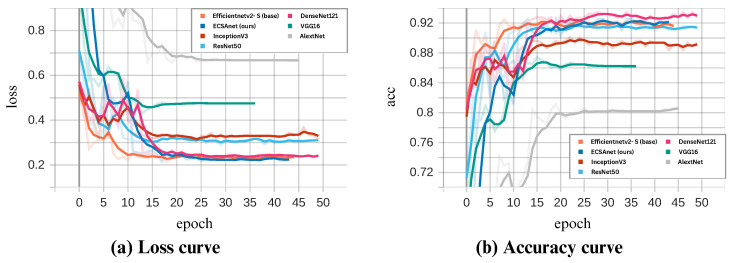
ECSAnet validation performance compared to other state-of-the-art models on the 100× magnification.

**Figure 17:**
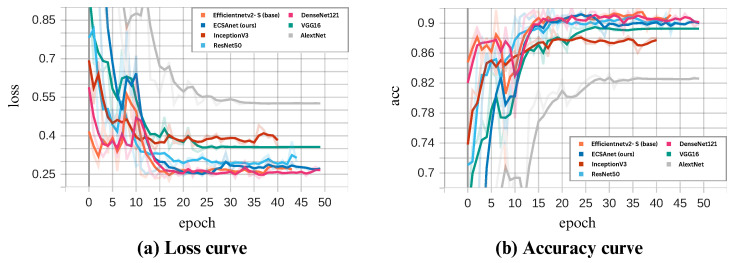
ECSAnet validation performance compared to other state-of-the-art models on the 200× magnification.

**Figure 18:**
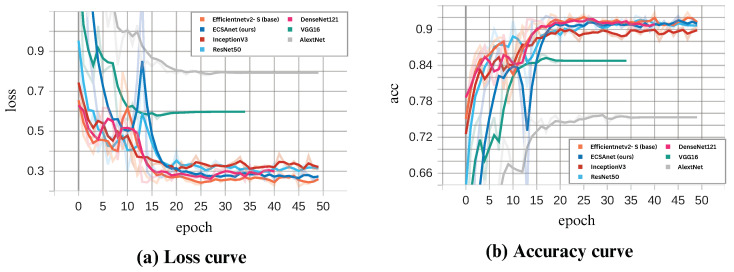
ECSAnet validation performance compared to other state-of-the-art models on the 400× magnification.

As a final evaluation of our results, we conducted a comparative analysis using six state-of-the-art models: AlexNet, DenseNet121, InceptionV3, ResNet50, VGG16, and lastly, the base model EfficientNetV2-S. All models were trained under the same settings as ECSAnet. In Figure 15, Figure 16, Figure 17 and Figure 18, the validation loss and accuracy of each model are compared with ECSAnet for each magnification factor: 40×, 100×, 200×, and 400×, respectively.

The testing metrics of the models are detailed in Table 11. Our proposed model outperformed the other models at the 40×, 100×, and 400× magnifications across most metrics, whereas at 200×, DenseNet121 showed a minimal increase in accuracy of 0.48% compared to our model. Notably, InceptionNetV3 rivaled our model in accuracy at 100× but with a lower precision. In addition, at 200× magnification, our model’s accuracy did not exceed that of the base model or ResNet50.

**Figure 19:**
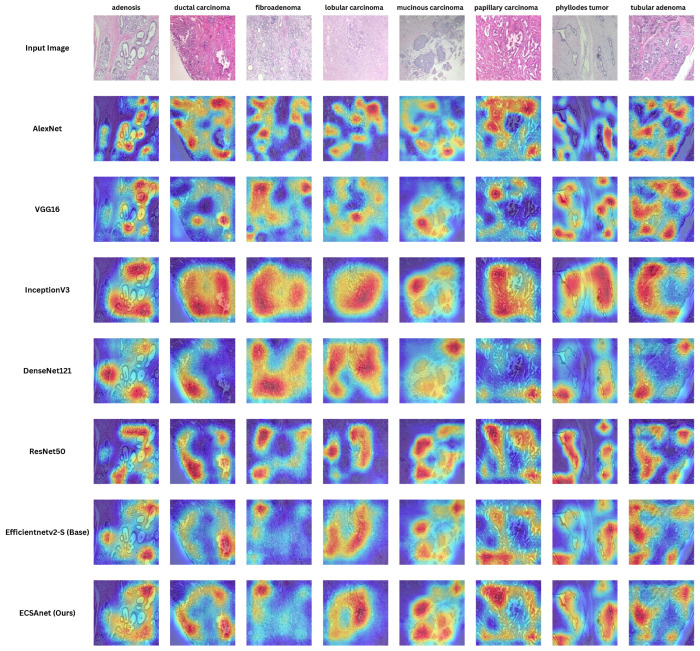
Grad-CAM visual explanations, illustrating the predictive focus areas for each class across models, descending vertically from the least accurate (AlexNet) to the most accurate (ECSAnet).

Furthermore, we compare the Grad-CAMs visual explanations for the same sample from each breast tumor sub-type across all models. In Figure 19, the Grad-CAM heat maps are presented. The decision-making interpretation of the models shows the most important regions of interest used for their decisions. With the incorporation of CBAM, the ECSAnet is capable of generating more accurate attention heat maps, making it more explainable than other models. Additionally, based on model performance, we assume that the features learned by ECSAnet were effective for correctly classifying the instances. However, the selection of regions made by each model needs to be assessed by domain experts to validate their accuracy and determine whether the ECSAnet model is truly effective.

## 5. Discussion

### 5.1. Main Findings

From our experiments, three characteristics become clear, upon which we base our main findings:

- The impact of magnification: A trend in our results showed that as the magnification factor increases, the model’s classification performance decreases; this observation can be seen in all the models that we have utilized. Similar results have been reported in other studies, such as Li et al.’s [25] binary classification and Boumaraf et al.’s [22] multi-class classification. Other studies have reported lower classification performance at 400× magnification specifically, such as [24,26]. Sheikh et al. [12] reported a counterpoint, saying that their approach performed better at higher magnifications. This contrast in findings indicates that the impact of magnification on classification accuracy is not yet fully understood and may be influenced by image processing techniques or specificities of model architectures, which require further investigation.
- Compatibility: Through our ablation study, we have found that the CBAM alone did not yield a performance improvement. When the CBAM was incorporated without additional FC layers, a decrease in model accuracy was noted. However, the model’s performance improved when we combined the CBAM with the implemented FC layers. This indicates that while the CBAM is adept at directing attention to salient features in the input data, the subsequent learning within the FC layers capitalizes on this refined focus to enhance the model’s accuracy. This suggests that there is a clear compatibility between our added components.
- Generalizability: Applying Reinhard stain normalization as a pre-processing step increased our model’s generalization capabilities on unseen test images, as confirmed in our ablation study. This enhancement aligns with the positive outcomes reported in studies that utilized the Reinhard stain normalization technique, such as [12]. However, it is important to note that the mentioned study did not quantitatively assess the extent to which stain normalization contributes to the improved generalization of their model. Our study fills this gap by evaluating the impact of stain normalization on model performance.

### 5.2. Model Strengths and Limitations

Our model tended to misclassify instances of various classes as DC. As observed from the lower specificity recorded for the DC class and decreased precision for other classes, including F, LC, MC, and PC. The misclassifications indicate intra-class similarities between the features of these classes and the DC class that led to confusion by the model.

As observed from the confusion matrices in Figure 12, the confusion between LC and DC was particularly notable across different magnifications. Specifically, the model misclassified LC images as DC, with six occurrences at 40× magnification and four occurrences each at 100× and 200× magnifications, decreasing slightly to three at 400×. Conversely, DC images were also often misclassified as LC, occurring once at 40×, increasing to four times at 100×, peaking at five times at 200×, and then, slightly reducing to four times at 400× magnification.

On the other hand, the model could classify A and TA classes with high accuracy and, in most cases, showed perfect scores. Moreover, it exhibited high specificity for all classes across magnifications, indicating the model’s ability to accurately identify true negatives and effectively minimize false positives.

## 6. Future Work

There are several ways to improve and expand our model’s capabilities. Future work may include conducting clinical trials to test ECSAnet’s effectiveness in real-world settings. Other key objectives involve enhancing classification accuracy for higher magnification settings and fine-tuning ECSAnet for benign vs. malignant binary classification. Moreover, we aim to develop the model’s ability to classify images at the patient level and achieve magnification-independent breast tumor multi-classification. To further validate ECSAnet’s robustness, we plan to test it on more extensive breast tumor histopathological datasets. Additionally, we will explore other attention mechanisms, experiment with different stain normalization techniques, such as Macenko and Vahadane, and investigate the effectiveness of various data augmentation methods, including patch generation and GANs.

## 7. Conclusions

Breast cancer remains one of the deadliest diseases worldwide, making the improvement of early diagnostic methods a critical area of research. In response to this challenge, we introduced the ECSAnet model architecture to enhance the accuracy and efficiency of multi-classification tasks in histopathological breast cancer image analysis. ECSAnet can extract discriminative breast cancer features efficiently along the spatial and channel dimensions using a CBAM attention mechanism with an increased learning capacity. The performance of ECSAnet has been evaluated on the BreakHis dataset at four magnification levels (40×, 100×, 200×, 400×). An ablation study has verified the importance of the added components in our model. Our model outperformed most state-of-the-art models in testing under the same training settings, attaining accuracies between 88.41% and 94.2% across magnification factors. The findings of this paper show positive outcomes that can help with the advancement of current clinical breast cancer diagnostic approaches, in addition to other cancers characterized by similar morphological cellular features [43].

## Figures and Tables

**Table 1:** ECSAnet architecture summary.

No.	Block	Channel Reduction Ratio	Expansion Factor	Kernel Size	Stride	No. of Layers	Output Channels
0	Conv	-	-	3 × 3	2	1	24
1	Fused-MBConv	-	1	3 × 3	1	2	24
2	Fused-MBConv	-	4	3 × 3	2	4	48
3	Fused-MBConv	-	4	3 × 3	2	4	64
4	MBConv+SE	0.25	4	3 × 3	2	6	128
5	MBConv+SE	0.25	6	3 × 3	1	9	160
6	MBConv+SE	0.25	6	3 × 3	2	15	256
7	CBAM	16	-	3 × 3 CAM, 7 × 7 SAM	1	4	256
8	Conv	-	-	1 × 1	1	1	1280
	+ AdaptiveAvgPool2D	-	-	-	-	1	1280
	+ Fully Connected	-	-	-	-	2	1024
	+ Fully Connected	-	-	-	-	1	8

**Table 2:** Class distribution of BreakHis.

Magnification	Benign				Malignant				Total
	A	F	PT	TA	DC	LC	MC	PC	
40×	114	253	109	149	864	156	205	145	1995
100×	113	260	121	150	903	170	222	142	2081
200×	111	264	108	140	896	163	196	135	2013
400×	106	237	115	130	788	137	169	138	1820
Sub-type total	444	1014	453	569	3451	626	792	560	7909
Type total	2480				5429				7909

**Table 3:** Training set after balancing and oversampling.

Magnification	Benign				Malignant				Total
	A	F	PT	TA	DC	LC	MC	PC	
40×	1812	1812	1812	1812	1812	1812	1812	1812	14,496
100×	1896	1896	1896	1896	1896	1896	1896	1896	15,168
200×	1881	1881	1881	1881	1881	1881	1881	1881	15,048
400×	1653	1653	1653	1653	1653	1653	1653	1653	13,224
Sub-type total	7242	7242	7242	7242	7242	7242	7242	7242	57,936
Type total	28,968				28,968				57,936

**Table 4:** Python libraries.

Library	Function Used
os	Handling file system, directory, and path operations.
time	Handling time-related tasks within code.
math	Computing basic mathematical operations.
torch	Creating the dataset classes and data loaders, loading the optimizer, criterion, and scheduler for model training, and logging model performance using Tensorboard.
torchvision	Access to V2 transforms for image data augmentations and loading pre-trained models.
PIL	Opening and manipulating images in the dataset.
collections	Counting the frequency of samples in dataset classes.
datetime	Access to the current date and time for model run logs.
uuid	Generating unique identifiers for model logs.
numpy	Handling data type conversions and mathematical operations.
sklearn [36]	Calculating different evaluation metrics and accessing data pre-processing tools.
seaborn [37]	Plotting confusion matrix.
matplotlib [38]	Plotting images and confusion matrix.
staintools	Reinhard stain normalizer.
grad-cam [39]	Model explainability methods to diagnose model predictions.

**Table 5:** Training hyperparameters.

Hyperparameter	Value
Learning Rate	0.001
Weight Decay	0.01
Batch Size	16
Number of Epochs	50
Loss Function	Cross-entropy loss
Optimizer	SGD
Learning Rate Scheduler	ReduceLROnPlateau
Early Stopping	Patience = 25 epochs

**Table 6:** ECSAnet classification results for each class on the 40× magnification test set.

Class	Precision (%)	Sensitivity (%)	Specificity (%)	F1 Score (%)	Support
A	100	100	100	100	13
DC	96	92	97.48	94	88
F	96	100	99.45	98	26
LC	71	94	96.86	81	16
MC	95	95	99.46	95	21
PC	93	87	99.48	90	15
PT	100	92	100	96	12
TA	100	100	100	100	16
Accuracy				94	207
Macro Average	94	95	99.09	94	207
Weighted Average	95	94	98.23	94	207

**Table 7:** ECSAnet classification results for each class on the 100× magnification test set.

Class	Precision (%)	Sensitivity (%)	Specificity (%)	F1 Score (%)	Support
A	100	92	100	96	12
DC	93	96	94.26	94	91
F	96	88	99.47	92	26
LC	78	78	97.95	78	18
MC	95	91	99.47	93	23
PC	94	100	99.49	97	15
PT	92	92	99.50	92	13
TA	100	100	100	100	15
Accuracy				93	213
Macro Average	93	92	98.77	93	213
Weighted Average	93	93	96.08	93	213

**Table 8:** ECSAnet classification results for each class on the 200× magnification test set.

Class	Precision (%)	Sensitivity (%)	Specificity (%)	F1 Score (%)	Support
A	92	92	99.49	92	12
DC	89	94	90.6	91	90
F	86	86	97.77	86	28
LC	71	71	97.37	71	17
MC	88	75	98.93	81	20
PC	100	86	100	92	14
PT	100	83	100	91	12
TA	93	100	99.48	97	14
Accuracy				88	207
Macro Average	90	86	97.82	88	207
Weighted Average	89	88	90.49	88	207

**Table 9:** ECSAnet classification results for each class on the 400× magnification test set.

Class	Precision (%)	Sensitivity (%)	Specificity (%)	F1 Score (%)	Support
A	100	100	100	100	11
DC	89	95	91.74	92	80
F	85	88	97.56	86	25
LC	77	67	98.28	71	15
MC	89	94	98.83	92	18
PC	100	80	100	89	15
PT	100	83	100	91	12
TA	85	85	98.86	85	13
Accuracy				89	189
Macro Average	91	87	98.16	87	189
Weighted Average	90	89	95.8	89	189

**Table 10:** Ablation results on the 40× magnification test set.

Model	Acc. (%)	Prec. (%)	F1 (%)	Jac. (%)	AUC (%)
ECSAnet+ No data augmentation	76.33	71.50	72.56	63.53	97.53
ECSAnet+ No balancing augmentations	93.24	93.82	93.35	87.98	99.39
ECSAnet+ No CBAM	92.75	93.58	92.96	87.46	99.67
ECSAnet+ No additional FC layers	91.79	92.25	91.93	85.69	99.70
ECSAnet+ No stain normalization	92.75	93.03	92.74	86.99	**99.85**
ECSAnet	**94.2**	**94.81**	**94.34**	**89.64**	99.62

Bold fonts indicate the best Values.

**Table 11:** ECSAnet performance on the test set compared to state-of-the-art models.

Model	Magnification	Acc. (%)	Prec. (%)	F1 (%)	Jac. (%)	AUC (%)
AlexNet	40×	82.13	84.42	82.50	70.81	98.46
	100×	78.40	80.36	78.52	64.93	97.70
	200×	77.29	79.82	77.96	64.92	96.28
	400×	74.07	74.36	73.67	59.79	95.91
DenseNet121	40×	91.30	92.15	91.53	85.09	**99.78**
	100×	92.02	92.18	92.05	85.75	99.44
	200×	**88.89**	**89.51**	**88.76**	**80.25**	**98.96**
	400×	86.77	87.38	86.30	76.63	98.85
InceptionNetV3	40×	90.82	91.89	91.09	84.21	99.60
	100×	**92.96**	92.97	92.92	**87.23**	99.20
	200×	87.44	87.63	87.32	78.38	98.10
	400×	84.13	84.58	83.24	72.57	98.13
ResNet50	40×	92.27	93.02	92.49	86.70	99.60
	100×	91.55	91.53	91.43	84.60	99.23
	200×	88.41	88.83	88.34	79.38	98.47
	400×	84.66	85.47	84.05	73.51	**99.25**
VGG16	40×	87.44	89.46	87.74	78.81	99.27
	100×	85.45	86.22	85.48	75.14	98.61
	200×	83.09	83.2	83.10	71.65	98.17
	400×	82.54	82.27	82.1	70.79	97.08
EfficientNetV2-S (base)	40×	93.24	93.94	93.41	88.07	99.70
	100×	90.61	91.02	90.68	83.55	99.44
	200×	88.41	88.38	88.32	79.73	98.28
	400×	87.30	87.89	87.09	77.53	99.09
ECSAnet (ours)	40×	**94.2**	**94.81**	**94.34**	**89.64**	99.62
	100×	**92.96**	**93.03**	**92.94**	87.19	**99.66**
	200×	88.41	88.6	88.32	79.65	98.34
	400×	**89.42**	**89.59**	**89.29**	**81.19**	99.1

Bold values indicate the highest values. Recall is omitted because it is equal to accuracy due to balanced image classes.

## Data Availability

The data presented in this study is available in the Breast Cancer Histopathological Database at: https://web.inf.ufpr.br/vri/databases/breast-cancer-histopathological-database-breakhis (accessed on 1 June 2024) [32].
